# Diagnostic Applications of Intraoral Scanners: A Systematic Review

**DOI:** 10.3390/jimaging9070134

**Published:** 2023-07-03

**Authors:** Francesca Angelone, Alfonso Maria Ponsiglione, Carlo Ricciardi, Giuseppe Cesarelli, Mario Sansone, Francesco Amato

**Affiliations:** Department of Electrical Engineering and Information Technology, University of Naples Federico II, 80125 Naples, Italy; francesca.angelone@unina.it (F.A.); alfonsomaria.ponsiglione@unina.it (A.M.P.); giuseppe.cesarelli@unina.it (G.C.); mario.sansone@unina.it (M.S.); framato@unina.it (F.A.)

**Keywords:** intraoral scanners, diagnostics, oral health, digital impression, 3D dental models

## Abstract

In addition to their recognized value for obtaining 3D digital dental models, intraoral scanners (IOSs) have recently been proven to be promising tools for oral health diagnostics. In this work, the most recent literature on IOSs was reviewed with a focus on their applications as detection systems of oral cavity pathologies. Those applications of IOSs falling in the general area of detection systems for oral health diagnostics (e.g., caries, dental wear, periodontal diseases, oral cancer) were included, while excluding those works mainly focused on 3D dental model reconstruction for implantology, orthodontics, or prosthodontics. Three major scientific databases, namely Scopus, PubMed, and Web of Science, were searched and explored by three independent reviewers. The synthesis and analysis of the studies was carried out by considering the type and technical features of the IOS, the study objectives, and the specific diagnostic applications. From the synthesis of the twenty-five included studies, the main diagnostic fields where IOS technology applies were highlighted, ranging from the detection of tooth wear and caries to the diagnosis of plaques, periodontal defects, and other complications. This shows how additional diagnostic information can be obtained by combining the IOS technology with other radiographic techniques. Despite some promising results, the clinical evidence regarding the use of IOSs as oral health probes is still limited, and further efforts are needed to validate the diagnostic potential of IOSs over conventional tools.

## 1. Introduction

Intraoral scanners (IOSs) are medical devices based on 3D measurement systems that are able to capture information on the shape and size of dental arches and to reproduce 3D models of the teeth and soft tissues of the oral cavity, thus allowing complete digitalization of the mouth anatomy [1,2]. Currently, commercially available IOSs are based on different noncontact optical technologies and principles [3]. IOS technology aims to overcome some of the major limitations of traditional dental impressions, which are time-consuming and are not comfortable for patients, and to implement a fully digitalized orthodontic workflow from 2D image acquisition up to 3D modeling and treatment planning [4].

IOSs, similarly to other scanners applied in different fields, project a light source onto the dental arches, including prepared teeth and implant scan bodies [5]. The images of the oral tissues, captured by the imaging sensors, are processed by the scanning software, which generates point clouds [6] that are then triangulated by the same software, creating a 3D surface model (mesh). The 3D tissue models are the result of optical impressions and are the digital alternative to traditional plaster models [4].

These devices are, therefore, born with the aim of creating a digital 3D model of the dental arches to replace the traditional dental impression. With technological innovations and suitable software systems, however, these intraoral devices could be used not only for the creation of a 3D digital model but also for diagnostic purposes. In some cases, software modules have been developed by the manufacturer of the scanner for the detection of pathologies based on optical technologies mounted on the device. In other cases, the diagnosis of some conditions and/or pathologies takes place using metrological analysis and 3D model inspection and processing software.

Oral health [7] is very often underestimated, but it is critical and contributes to total health and well-being. Recent research indicates that poor oral health affects overall health and that some systemic diseases can affect oral health [8]. The most prevalent oral disease is dental caries, which is preventable, and significant efforts have been made to control this condition. Other diseases and conditions are much less common but are serious and sometimes even life-threatening: oral precancer and cancer [9], oral manifestations of HIV and AIDS [10], developmental disorders [11], and tooth fluorosis [12]. The potential to integrate the need for Computer-Aided Design and Computer-Aided Manufacturing (CAD/CAM) with diagnostic capability into a single device without resorting to additional, even invasive, tools, can greatly increase the benefits for the patient and clinician.

In previous years, quantitative methods for detecting and monitoring the course of oral cavity diseases have been introduced. Very often, radiographic techniques or visual-tactile methods [13,14,15] are used for the detection of these pathologies with limitations related to intra- and interclinical variability. The introduction of digital into clinical practice can provide an additional tool, not only for dental impressions and orthodontic or implant treatment planning, but also for diagnosing structural and functional disorders directly at the chairside, easing the clinical workflow for both the clinician and patient. It is evident that the introduction of tools based on optical principles is also becoming increasingly prevalent in diagnostics [16,17,18], as reliable quantitative results are sought to support clinical decision-making.

The purpose of this systematic review is to explore the potential of IOSs, which are routinely used for the reconstruction of 3D dental models for orthodontic treatments and planning and can also be implemented beyond the scope of orthodontic interventions, thus providing alternative diagnostic tools for the detection of oral cavity pathologies/anomalies (e.g., caries, dental wear, periodontal diseases, oral cancer, infections) to traditional methods or available gold standards (e.g., radiographic modalities). After defining a PICO Statement (Population, Intervention, Comparison, and Outcome Statement), the scientific literature was explored, screened, and analyzed with a specific focus on those documents dealing with diagnostic applications of IOSs in the field of oral health. A synthesis and analysis of the included studies was carried out by taking into account different characteristics, information, and items extracted from the collected documents, namely the type of device, the technical characteristics of both the hardware and software components of the adopted IOS, the objective and type of study, and the unique diagnostic application. The main diagnostic fields where IOS technology applies were highlighted, the most promising results were summarized and commented on by underlining the pros and cons of the proposed technological solution and related diagnostic application, and the future directions in the use of IOSs as diagnostic tools in clinical practice were critically discussed.

## 2. Materials and Methods

### 2.1. Eligibility Criteria

Systematic reviews allow us to evaluate and summarize studies considering predetermined eligibility criteria to answer a specific research question. After defining the PICO Statement shown in Table 1, PRISMA (Preferred Reporting Items for Systematic Reviews and Meta-Analyses) criteria were applied [19].

The main research question is focused on investigating the potential of IOSs as alternative diagnostic tools for the detection of oral cavity pathologies compared to traditional methods.

Only studies, as properly framed in the objective of this review, taking into the following aspects were considered eligible:

- The use of intraoral scanner devices in the oral health diagnostics field;
- The assessment of performance in the detection of oral cavity pathologies (e.g., caries, dental wear, periodontal diseases, oral cancer, infections);
- Clear description of the diagnostic workflow;
- Comparison with a reference method or gold standard.

The literature screening was carried out by considering those applications of IOSs falling in the general area of detection systems for oral health diagnostics (e.g., caries, dental wear, periodontal diseases, oral cancer). Those works mainly focused on 3D dental model reconstruction for implantology or orthodontics or prosthodontics were excluded. In particular, the following exclusion criteria were defined:

- Papers dealing with the technical evaluation of the accuracy and precision of intraoral scanners in reconstruction systems to achieve 3D digital mapping of the oral cavity;
- Papers exploiting digital models obtained from intraoral scanning to fabricate prostheses or to replace prostheses already implanted in the patient’s mouth.

### 2.2. Information Sources and Search Strategy

A comprehensive search of the online databases PubMed, Scopus and Web of Science was conducted in English using indexed and free-text words produced in the past five years (January 2018–December 2022). Since the development of digital technology in the dental field has been limited to the last few years, the research was limited to the last five years. The following logical search query was used: (“intraoral scanner” AND “diagnos*”)

The query, properly adapted to each specific database accessed, was used to check the following textual fields of the documents: title, abstract, and keywords.

In addition, non-English articles, duplicates, conference papers, reviews, book chapters, and papers not available in the first phase of research were excluded.

### 2.3. Selection Process

The study selection process was carried out according to the PRISMA flowchart. During the screening of the databases, the duplicates were removed, and the type of publication and language were investigated, as described in the previous paragraph. Subsequently, an analysis by titles and abstracts was carried out, excluding papers that clearly did not define the diagnostic purpose of the intraoral scan. The full-text of the selected papers was then examined to verify their eligibility according to the previously defined criteria.

Abstract and full-text screening was conducted taking into consideration that this review aimed to shed light on those applications of IOSs falling in the general area of detection systems for oral health diagnostics (e.g., caries, dental wear, periodontal diseases, oral cancer), thus not strictly pertaining to the implantology (e.g., guided implant surgery), orthodontics (e.g., aligners, cast production, virtual patient, digital impression), or prosthodontics (e.g., digital smile design, dental restoration) fields.

The in-depth analysis of the full-texts has made it possible to identify the main diagnostic fields in the oral health area in which it is possible to use intraoral scanning, once again excluding all those articles that go beyond the scope of this systematic review.

### 2.4. Data Collection

The data collection was carried out based on a customized Microsoft Excel form, mainly collecting the following information: authors, title of the article, year of publication, Digital Object Identifier (DOI), and abstract. The articles were divided equally among the reviewers. Possible doubts about inclusion/exclusion and their categorization were discussed until an agreement was reached.

From the subsequent screening of full-text articles, it was possible to collect information on the type of IOS used, the use of in vivo or in vitro models, diagnostic applications in the oral health area, and software used. No contact with the authors of the manuscripts for complementary information was necessary.

### 2.5. Data Item Clustering

The studies were initially divided considering the diagnostic application field by identifying the diagnostic application for which the IOS would be most used. Subsequently, the number of studies using in vivo or in vitro models was investigated. The main types of IOS used in the main diagnostic fields were then analyzed by investigating the optical characteristics that allow the identification of certain conditions or pathologies of the oral cavity. Finally, the articles were clustered according to the software used, thus making it possible to evaluate the most-used software in the dental field.

### 2.6. Risk of Bias

To reduce any bias, the Excel form used by the reviewers to collect information was agreed on in advance, the names of the different devices were standardized, and a dedicated comments section was set up to allow any concerns to be reported.

### 2.7. Synthesis Methods and Analysis of the Results

From the previously described clustering, two main diagnostic applications in the oral health area have been identified for which the use of an IOS has shown promising results, in addition to other less investigated applications in the literature. The analysis is graphically represented using the Excel graphical tools, mainly through histograms and pie charts, which better highlight the numerical distribution in the different categories. In addition, tables are used to facilitate a comparison between the various devices.

## 3. Results

### 3.1. Main Findings

**Figure 1:**
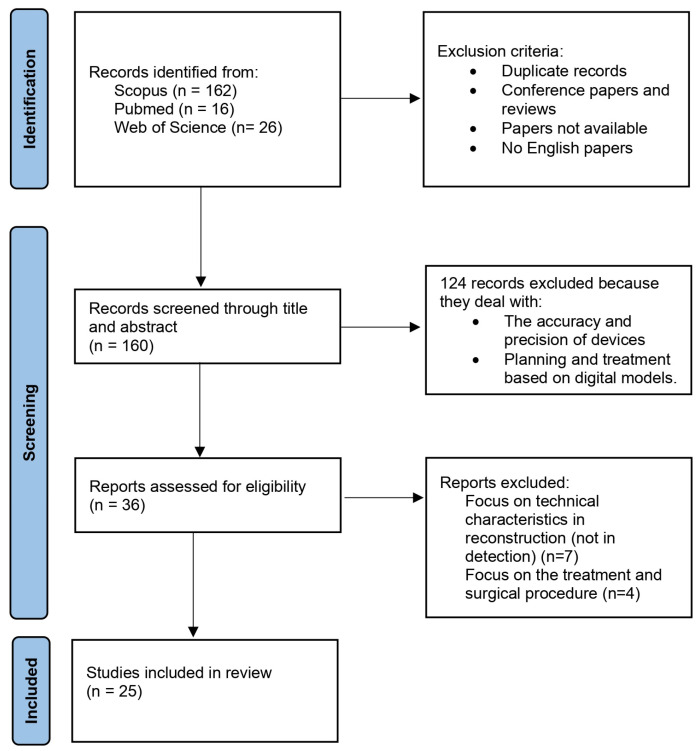
PRISMA review workflow.

Only those articles introducing the use of an IOS for diagnostic/detection purposes in the oral health field, as defined in the methodological section of this work, were included. Based on the eligibility criteria, the analysis was conducted by first analyzing papers by title and abstract and then by full-text. The PRISMA workflow is shown in Figure 1.

**Figure 2:**
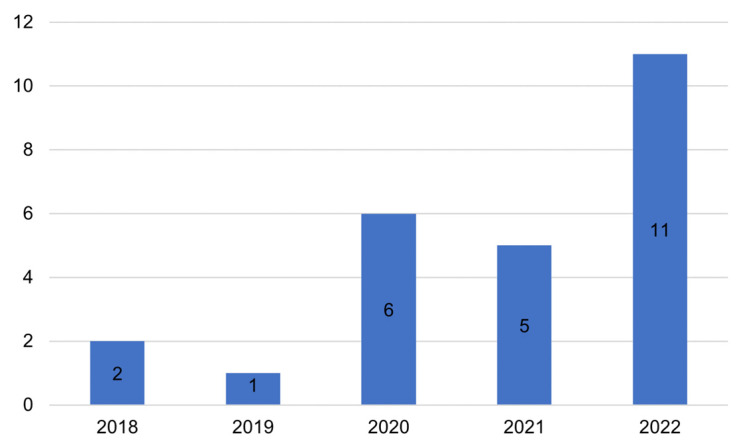
Distribution of the included studies across the years. The number of studies is reported on the y-axis.

The search was limited to the last 5 years, from 2018 to 2022, and the papers included were mainly concentrated in 2020 and 2022, as shown in Figure 2.

The papers were analyzed according to several categories: aim of the study; type of IOS used; use of in vivo or in vitro models; diagnostic application; software used; and results of the studies. Table 2 summarizes these fields for each paper analyzed.

Table 3 also describes the fields defined for the papers excluded from the full-text analysis to better clarify the difference.

Unlike the included studies, where the use of intraoral scanners in the diagnosis of pathologies of the oral cavity was discussed, the excluded studies mainly concerned the evaluation of the technical performance in the reconstruction (and not the detection) of the digital model compared with traditional methods and the description of digital planning and design flows of orthodontic and implantological treatments, etc. The purpose of the review was, therefore, mainly to evaluate the possibility of using scanners commonly used in the digital reconstruction of dental models for the diagnosis and evaluation of oral health, excluding applications that base their efficiency on a good reconstruction of the 3D model, and to conduct planning on them. In particular, evaluations of the reconstructed digital model were excluded, as they made mere comparisons of the technical parameters of the 3D model obtained with intraoral scanners and with traditional methods. In particular, Ferraro et al. [44] and Akyalcin et al. [45] compared the digital model obtained with an intraoral scanner (TRIOS (3Shape, Copenhagen, Denmark) in the first case and the Cadent iTero scanner (Align Technology, San Jose, CA, USA) in the second case with that obtained by starting from a CBCT (ProMax 3D Mid; Planmeca, Roselle, Ill) in the first case and a CS 9300 unit (Carestream Health, Atlanta, GA, USA) in the second case with differences being not clinically detectable for orthodontic applications. Fraile et al. [46] described a cross-sectional study to compare the interocclusal contact records obtained with three different digital methods (intra- and extraoral digital scanners and the T-Scan III system) with the conventional method (articulated paper), demonstrating good reliability of the scanners compared to the gold standard. Kirschneck et al. [47] compared the intraoral scan with an extraoral one and the polyether impression (reference), demonstrating better reliability of the extraoral 3D models.

In other cases, the purpose of the excluded papers was to plan an implantological, endodontic, restorative, or esthetic treatment. Davidovich et al. [48,49] presented an innovative therapeutic approach for endodontically treated teeth and treatment for the hypomineralization of molar incisors (MIH) in children using a digital workflow with an IOS and CAD/CAM for restoration, addressing the uncooperative behavior of children and enabling the preservation of the tooth structure and long-lasting restoration. Revilla-Leon et al. [50,51] and Park et al. [52] instead presented a digital workflow for esthetic rehabilitation (e.g., in case of diastema) using a combination of facial and intraoral scanners and additive manufactured (AM) clear silicone indices. DuVall [53] used an intraoral scanner and milling unit to fabricate a CAD/CAM radiographic and surgical guide for use with a Cone Beam Computed Tomography system. Ahmed et al. [54] tested the use of a digital approach to lengthen crowns, reducing the likelihood for the need for postsurgical modifications and shortening the treatment time.

### 3.2. Oral Conditions Diagnosed Using an IOS

**Figure 3:**
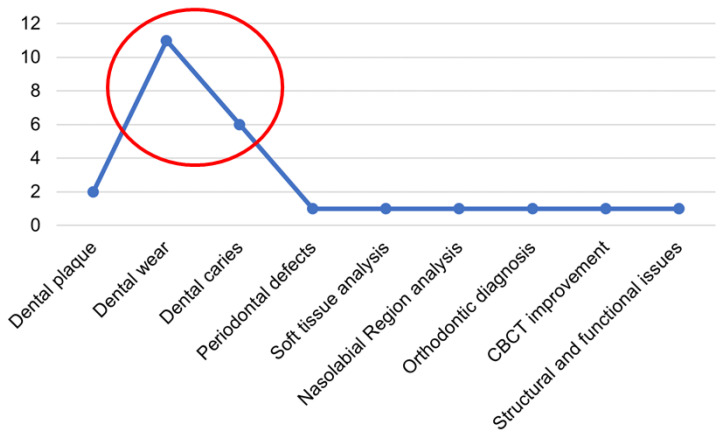
Distribution of the main oral health pathologies/anomalies addressed in the included studies. The number of studies focusing on each pathology/anomaly is reported on the y-axis. The red circle highlights the two oral health topics occurring the most in the included studies (indeed, dental wear and dental caries diagnostics are addressed in almost 70% of the included studies).

Among the papers analyzing the diagnostic purpose, most investigated the ability to assess dental enamel wear, followed by articles discussing caries detection and dental plaque evaluation, as shown in Figure 3.

Other articles included cover the evaluation of functional and structural problems, the evaluation of periodontal defects, soft tissue analysis, orthodontic diagnosis, and analysis of the nasolabial region. Finally, one paper [25] discusses the improvement of the Cone Beam Computed Tomography (CBCT) technique using intraoral scanners.

It is interesting to note that most of the papers found did not investigate the diagnostic purposes of the IOS (so they were excluded from the analysis) but mainly dealt with the use of an IOS for the evaluation of 3D models obtained through device use and treatment planning with digital flow.

### 3.3. In Vivo and In Vitro Studies

We intended to investigate the use of in vivo or in vitro models in the analyzed papers. It was noted that, out of the total number of papers included, the distributions of the use of in vivo versus in vitro models were almost the same (12 articles used in vitro models and 13 used in vivo models). The difference was more significant when considering only the articles dealing with the evaluation of dental wear. In this case, the use of in vitro models was predominant (64%). This is because, in many cases, dental wear is induced through chemical agents or mechanical techniques, and therefore, in vivo models are impractical.

### 3.4. Analysis of the Main Commercial IOS

Wanting to analyze the application of intraoral scanners in diagnostics, we started with a market analysis to understand which are the main IOSs on the market and to understand the technology behind 3D reconstruction. The main features of the most popular scanners on the market are shown in Table 4. In particular, data were collected by investigating the sites of the manufacturers and previous reviews comparing some intraoral scanners [55,56,57,58,59,60,61,62] and by meetings with dentists who use these devices. The survey with clinicians showed that such devices are still viewed with distrust by some who still prefer traditional impressions, and those who choose such devices for clinical practice tend to go for the top-of-the-line products from the different manufacturers. In terms of technology, almost all of them are based on 3D reconstruction technology based on structured light, that is, imaging and capturing the deformation of a projected light pattern that is deformed on the dental arch. 

### 3.5. Technologies for Oral Diagnostics

**Figure 4:**
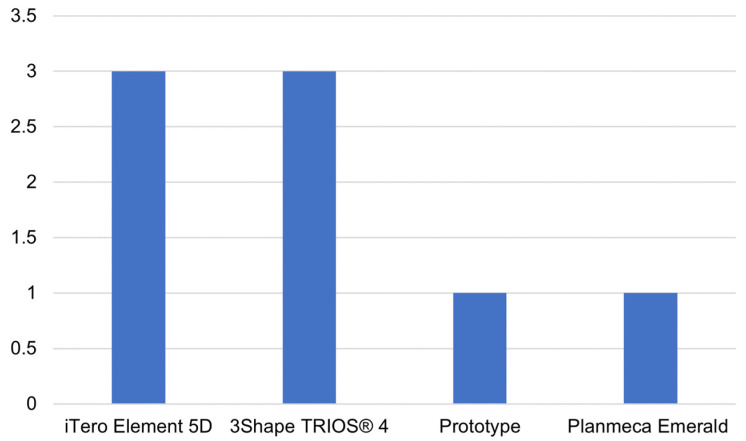
Main intraoral scanners used for dental caries detection. The number of studies adopting each specific scanner for caries evaluation is reported on the y-axis.

**Figure 5:**
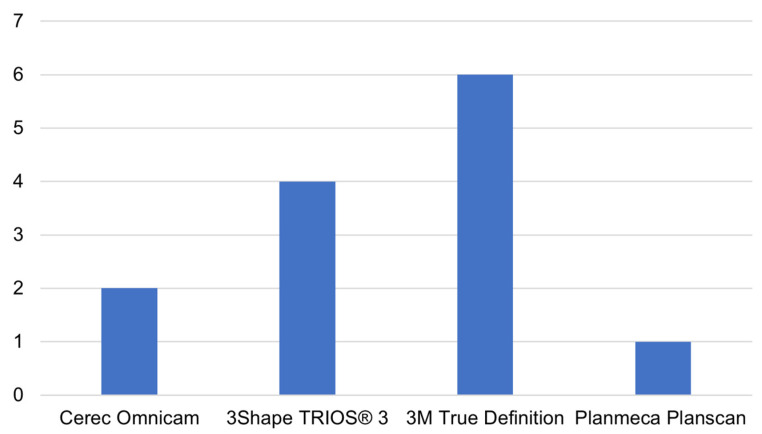
The main intraoral scanners used for dental wear evaluation. The number of studies adopting each specific scanner for dental wear evaluation is reported on the y-axis.

It is of interest to assess which scanners are most widely used in the different diagnostic areas defined in Section 3.2. By analyzing the scanning devices used for diagnostic purposes in the main diagnostic fields found, it was possible to obtain the distribution shown in Figure 4 and Figure 5.

As expected, in caries detection, the most widely used scanners are those that, in addition to scanning for the creation of the 3D model of the dental arches, have integrated technology for caries detection. In particular, iTero Element 5D (Align Technology, Inc., Tempe, Arizona) implements NIRI technology, which uses near-infrared (light with a wavelength of 850 nm) to highlight areas of demineralization with a bright region, allowing even interproximal caries to be identified. TRIOS 4 (3Shape A/S, Copenhagen, Denmark) has a built-in fluorescent technology for caries detection, mainly on the occlusal surfaces. The only other scanner on the market that has caries detection technology is Emerald S (Planmeca, Helsinki, Finland). The latter has a dedicated tip that implements transillumination-based technology, allowing it to detect supragingival proximal carious lesions. Michou et al. [35] developed an intraoral scanner prototype that emits light at 415 nm, thus taking advantage of fluorescent light.

To assess dental wear, on the other hand, the most widely used intraoral scanner is certainly True Definition (3M, St. Paul, MN, USA), followed by TRIOS 3 (3Shape A/S, Copenhagen, Denmark), Cerec Omnicam (Dentsply Sirona, Bensheim, Germany) and Planscan (Planmeca, Helsinki, Finland).

The intraoral scanners used in the other identified diagnostic fields are summarized in Table 5.

### 3.6. Three-Dimensional Model of Evaluation Software

**Figure 6:**
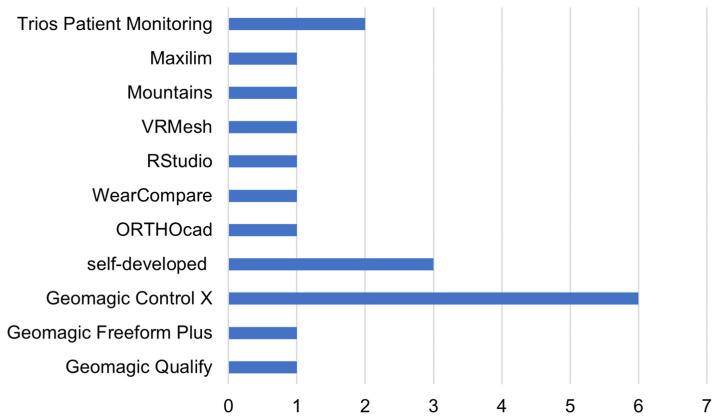
Main software used for the assessment of 3D dental models. The number of studies adopting each specific software tool is reported on the x-axis.

After obtaining the 3D model of the dental arches using intraoral scanning devices, a series of quantitative assessments must be made on the model itself to arrive at a diagnosis or assessment of oral conditions. Appropriate software was used to make these assessments in many articles reviewed, the distribution of which is shown in Figure 6. The most widely used software is Geomagic Control X (3Dsystems, Darmstadt, Germany), as shown in Figure 6.

**Figure 7:**
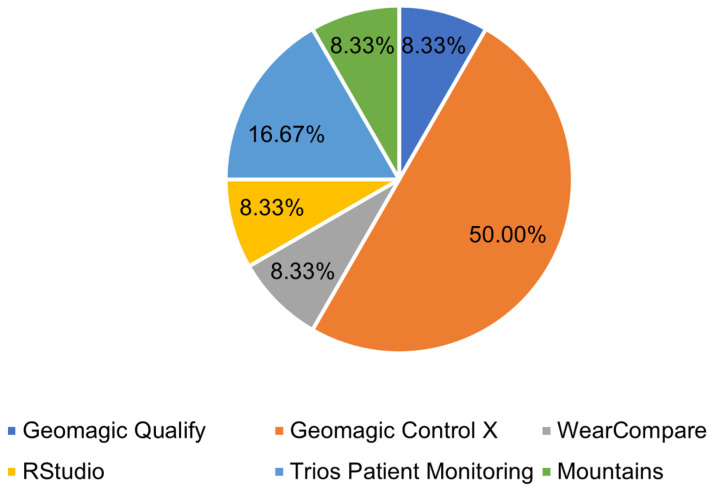
The main software and tools used for dental wear evaluation.

This prevalence was even more pronounced when considering only dental wear cases, where half of the papers made model comparisons using Geomagic Control X software, as shown in Figure 7.

In fact, most evaluations conducted on dental wear were based on a comparison between 3D models before and after wear, where the use of 3D inspection and metrology software is required. Other metrology software used in [30,41] were Mountains (Digitalsurf, Besançon, France) to measure step height on a freeform surface by comparing four workflow analysis techniques and Geomagic Qualify to quickly and easily overlay, evaluate, and report deviations between designed and built parts. In addition to metrology and 3D model comparison software, point cloud processing software was also used, such as VRMesh (VRMesh studio VirtualGrid), used in [32] to evaluate scarring and asymmetry of the upper lip in surgically managed cases of unilateral cleft lip and cleft palate (UCLP). In [25], a segmented scan was conducted using Geomagic Freeform Plus (3Dsystems, Darmstadt, Germany), a 3D Design and Sculpting Software, to join the models of the arch pieces together. RStudio, with the “molarR” package [63], was also used to produce topographic parameters on which the occlusal tooth wear assessment was based in [37].

In other cases, specific software referring to the dental field was used, such as WearCompare (leedsdigitaldentistry.com, accessed on 20 June 2023) [64], a purpose-based software specifically produced to assess tooth wear; TRIOS Patient Monitoring (3Shape A/S, Copenhagen, Denmark), proprietary software from the same company that produces the TRIOS IOS, which allows the visual recording of changes over time; OrthoCAD (Cadent, Inc., San Jose, CA, USA), proprietary software from the same company that produces the iTero scanner, which allows the analysis of dental arch models, enabling measurements of the width of dental arches, such as in [22], where the authors evaluated the effect caused by removable appliances over a period of 10 months in children with malocclusion; and Maxilim software (V2.3.0, Medicim NV, Mechelen, Belgium), which is used to align the IOS models. Additionally, self-developed software was used to address specific needs, such as the evaluation of dental plaque in 3D models [20], the assessment of teeth [21], and the identification of structural and functional problems along with a carefully designed OCT hardware prototype [42].

## 4. Discussion

We systematically reviewed the possible applications of intraoral scanners in the diagnostic field. It was found that the main diagnostic application area of IOSs is the evaluation of dental wear. Dental wear affects overall health and well-being by involving structural problems (support structures and loss of the vertical dimension of occlusion) and functional problems (increased tooth sensitivity, chewing, temporo-mandibular joint dysfunction, headaches, etc.) [65]. The evaluation of tooth wear can, therefore, be very useful for preventing various disorders. Most of the papers analyzed were based on in vitro studies, as wear is induced either with chemical agents or mechanically, simulating factors that may influence tooth wear (habit-, diet-, and musculature-specific factors). The accuracy of scanners when identifying changes before and after exposure to these factors was then evaluated with 3D metrology software, as described in Section 3.6.

In particular, Kühne et al. [30] performed a comparison between the reference model acquired by noncontact white light profilometry and 3D models acquired with different scanners (TRIOS 3, Cerec Omnicam, True Definition Scanner) at three different stages of wear simulated with a diamond bur using the Geomagic Qualify 2012 version, confirming the ability of IOSs to evaluate dental wear, even considering an imprecision level of plus or minus 20 µm with respect to the profilometry. With chemically induced wear and using only the True Definition Scanner, Kumar et al. [40] concluded that while IOSs are promising for the assessment of dental wear, they may not capture small variations. In fact, after 10 min of exposure, a volume change of −0.45 mm^3^ (±2.59) was noted with a high standard deviation that was too large to perform an accurate volumetric analysis, due to the dimension of the triangles created by the True Definition Scanner being 50 µm. Ille et al. [34], after three periods of tooth soda exposure, the acquisition of the arches with the Planmeca scanner, and using Geomagic Control X as the metrology software, concluded that the latter scanner is capable of detecting even small variations, detecting an average of 65 µm of dental tissue over the course of 19 h of exposure. Michou et al. [38] also evaluated wear after citric acid exposure session using TRIOS 3 and related TRIOS Patient Monitoring software, confirming the potential of this IOS for assessing tooth wear. Along with TRIOS Patient Monitoring, which is useful for assessing depth loss (mm), Machado et al. [23] used also WearCompare software to obtain the volume loss (mm^3^) and area loss (mm^2^), demonstrating that there are strong correlations between depth (mm^2^) and time (r = 0.9993 *p* < 0.0001), volume (mm^3^) and time (r = 0.9968, *p* < 0.0001), and area (mm^2^) and time (r = 0.9475, *p* = 0.0003). In order to adequately assess tooth wear, it is important to define indices that can be quantitatively and objectively recognized. To date, there are indices for the visual assessment of tooth wear (such as the Basic Erosive Wear Examination (BEWE) [66]) which, although useful, are subject to bias depending on the operator’s clinical experience. In fact, Travassos da Rosa Moreira Bastos et al. [31] performed a study to assess intra- and interobserver concordance based on scans taken one month from baseline, concluding that the visual evaluation based on intraoral scanning results in a lower bias with a moderate agreement for the intraoral scanner analysis (K = 0.595) using the Kappa test. However, to make the assessment more objective, Alwadai et al. [37] aimed to investigate the effectiveness of occlusal (as they are highly susceptible to wear) topographical analyses to assess the progression of simulated wear. Abrasion, in this case, was mechanically simulated using silicon carbide grind papers, and the evaluation was based on a combination of the digital impression scanner and dental topographic analysis parameters (Slope, Relief, RFI, and OPCr) calculated in RStudio with the “molaR” package. These topographic attributes were expected to decrease as induced wear increases, which was observed (e.g., the slope varied between 54.6 (±4.3) and 46.6 (±6.4) when the wear increased from 0 mm to 1.5 mm).

In vivo analyses of tooth wear are evaluated on a longer time basis. For example, in [26,36], wear was evaluated relative to baseline after 6 months and 1 year, obviously having weaker variations and taking respectively the visual evaluation and the microCT as references. Time is further extended (12 and 24 months) when wear is evaluated on lithium disilicate implant crowns and their enamel antagonists, as conducted by Stück et al. [21].

Another clinical area where scanners can provide good diagnostic results is caries detection. In this field, scanners with integrated caries detection systems based on noninvasive optical systems, described in Section 3.5, are already under development and are spreading on the market. The first scanner with integrated caries detection technology was TRIOS 4, which integrates fluorescence technology, whose diagnostic reliability was investigated in many of the articles analyzed [48,49,50]. Even near-infrared lights have the ability to locate carious lesions, and this technology has been implemented in iTero Element 5D. In this case, a greater ability to detect interproximal caries was found [27,29], even in comparison with radiography, which is considered the gold standard for these evaluations [15]. Schlenz et al. [29] made a comparison between the three previously mentioned scanners and traditional methods, finding the greatest reliability for diagnosing occlusal caries in permanent teeth with Planmeca Emerald S, which is based on transilluminescence technology. Another relevant study was carried out by Michou et al. [35], who developed an intraoral scanner prototype that emits light at 415 nm, thus taking advantage of fluorescent light. They compared it with visual–tactile, radiographic, and histological assessments of caries and showed the promising performance of fluorescent light.

Dental caries and periodontal diseases are plaque-associated conditions, so it is also important to define appropriate methods for assessing this condition. In clinical practice, plaque levels are recorded chairside using index systems that calculate the amount of plaque, despite not showing very robust results. Two of the analyzed studies aimed to investigate whether plaque can be reliably detected, quantified, and monitored on 3D models of dental arches acquired by intraoral scanners. In the first case [20], this was conducted by measuring the amount of plaque with planimetric measurements using appropriate especially designed software; in the second case [24], it was conducted by comparing visual assessments performed chairside and with the 3D model. In both cases, the IOS was proven to be an adequate tool for measuring plaque.

Intraoral scanners have also proven to be excellent tools for making assessments of structural problems in the oral cavity, such as quantifying changes in the width of the dental arches after treatment, e.g., with removable braces in children with malocclusion (orthodontic diagnostics) [22]; in the external regions, such as the evaluation of scarring and asymmetry of the upper lip in surgically managed cases of unilateral cleft lip and cleft palate (UCLP) [32]; and overall in the cranial region, creating hybrid images resulting from the crossing of several diagnostic sources, such as Cone Beam Computed Tomography (CBCT) of the skull with a 3D model of dentition derived from a scanner [25]. In the context of diagnosing functional and structural problems, there is the interesting study by Eom et al. [42], who designed and evaluated the performance response of a three-dimensional (3D) intraoral scanning probe based on optical coherence tomography (OCT), showing the ability to reconstruct both the structure and function of human teeth. The ability of intraoral scanners to allow the evaluation of structural problems is not limited to the dental structure but was also investigated by Deferm et al. [43] for the analysis of soft tissues, in terms of shape, color, and curvature.

## 5. Conclusions

From the analysis of the different papers, it emerged that the use of intraoral scanning probes represents a promising approach that could be used in the fields of orthodontics and implantology, as well as in various diagnostic fields, for structural evaluations, such as the evaluation of dental erosion, which emerged from this review as the most promising field. In the detection of caries, scanners are already considered valid and established tools on the market and are used in dental clinics. The differences between the scanners are mainly due to the 3D imaging principle, the different wavelengths used, the image acquisition principle, and the scanner wand. More research is needed to test their performance levels in the context of their differences, which appear to be established only in the case of dental caries.

In conclusion, there is a wide range of possible diagnostic applications, but the cases in which dental impression analysis can be considered the clinical standard are limited, due to the still-necessary validation compared to traditional methods.

However, the results of the studies analyzed encourage the idea that, in the near future, also thanks to further technological innovations, there is the possibility of using intraoral scanners as diagnostic devices in clinical practice, providing quantitative parameters on which to base clinical decision-making, with advantages for both the clinician and the patient.

## Figures and Tables

**Table 1:** Different components of a PICO statement for the current study.

PICO Facets	Considerations
Patient (P)	Patients with oral cavity pathologies (e.g., caries, dental wear, periodontal diseases, oral cancer, infections)
Intervention (I)	Detection of oral cavity pathologies by means of an intraoral scanning device
Comparison (C)	Traditional methods or gold standards for the diagnosis/detection of the specific oral cavity pathology (e.g., radiographic modalities)
Outcome (O)	Assessment of the performance of intraoral scanners in the diagnosis/detection of pathologies of the oral cavity compared with reference methods

**Table 2:** Summary of the items and categories examined for the included papers.

First Author (Year)	Aim of the Study	Type of IOS	Product Name	Type of Study	Diagnostic Application	Software Used	Main Results
Jung et al. (2022) [20]	To investigate whether images from 3D intraoral scans are also suitable for valid planimetric plaque measurements and monitoring	Commercial	CS3600 Carestream	In vivo	Dental plaque	Self-developed software (Julia (version 1.6.28)	Planimetry, using images from the 3D intraoral scan seems to be a suitable tool for recording and monitoring dental plaques.
Stück et al. (2022) [21]	To evaluate the difference between the dental wear in implant/enamel and enamel/enamel contacts	Commercial	CEREC omnicam	In vivo	Dental wear	Python Software Foundation, Wilmington, Delaware, United States of America, version 3.5.2; Blender Foundation, Amsterdam, the Netherlands, version 2.7;	Intraoral scanning and computer analysis showed that the two-years wear ratios for enamel/enamel and enamel/lithium disilicate implant crowns did not differ significantly.
Pałka et al. (2022) [22]	To evaluate the impact of removable appliances used over a 10 month period on growth changes in children with malocclusions	Commercial	iTero	In vivo	Orthodontic diagnosis	OrthoCAD (software version not specified-https://www.itero.com/it/education-and-support/software-downloads; accessed on 26 June 2023)	The use of removable appliances in children with a narrowed maxillary transverse dimension contributes to offsetting growth changes in comparison to children with normal occlusion.
Machado et al. (2022) [23]	To evaluate the potential of an IOS to monitor dental wear using distinct quantitative measurement metrics	Commercial	TRIOS 3	In vitro	Dental wear	WearCompare (software version not specified-https://leedsdigitaldentistry.com/wearcompare/; accessed on 26 June 2023); 3Shape TRIOS^®^ Patient Monitoring (software version not specified-https://www.3shape.com/en/software/trios-patient-monitoring; accessed on 26 June 2023)	This IOS is a potential clinical tool for detecting and quantitatively monitoring early and advanced erosive tooth wear.
Giese-Kraft et al. (2022) [24]	To investigate whether plaque can be reliably visualized on 2D and 3D images captured with digital intraoral imaging devices	Commercial	CS3600 Carestream	In vivo	Dental plaque	N/A	Amounts of plaque can be reliably detected and monitored on 2D images from an intraoral camera and on 3D images from an intraoral scanner.
Lee et al. (2022) [25]	To evaluate the potential for improving dentition imaging with CBCT scans using an IOS	Commercial	CS3600 Carestream e i700 Medit	In vivo	CBCT improvement	Geomagic Freeform Plus (software version not specified-https://oqton.com/geomagic-freeform/; accessed on 26 June 2023)	The virtual skull dentition hybrid image obtained from intraoral scanners will be clinically useful.
Metzger et al. (2022) [15]	To compare the detection of proximal caries with NIR versus BWR	Commercial	iTero Element 5D	In vivo	Dental caries	N/A	NIR was more sensitive than BWR for detecting early caries.
García et al. (2022) [26]	To analyze the sensitivity and specificity of the IOS for measuring dental wear and to evaluate patients’ satisfaction	Commercial	3M™ True Definition	In vivo	Dental wear	Geomagic™ (software version not specified-https://www.3dsystems.com/software; accessed on 26 June 2023)	The data show good levels of specificity and sensitivity, and the participants also show a high degree (40,9%) of satisfaction.
Sobral et al. (2022) [27]	To define a protocol to evaluate the best strategy for diagnosing primary caries lesions located in the interproximal region among visual clinical examination, IOS, and BWR	Commercial	iTero Element 5D	In vivo	Interproximal caries	N/A	Through the protocol described, clinicians will be able to assess whether there will be any difference in effectiveness between the selected diagnostic methods.
Michou et al. (2022) [28]	To assess the validity of an IOS system featuring NIR transillumination to aid in the detection of proximal caries lesions	Commercial	TRIOS 4	In vitro	Interocclusal caries	N/A	IOS system featuring NIR transillumination and DIAGNOcam showed an overall good diagnostic performance.
Schlenz et al. (2022) [29]	To investigate new caries diagnostic tools, including three IOS, and compare them to established diagnostic methods	Commercial	1)iTero element 5D;2)TRIOS 4;4)Planmeca Emerald S	In vitro	Proximal and occlusal caries	N/A	Caries diagnostics with intraoral scanners seem to be interesting tools that should be further investigated in clinical studies.
Kühne et al. (2021) [30]	To investigate whether IOSs are suitable for wear measurement compared to WLP	Commercial	Cerec Omnicam AC (OC), TRIOS 3 (Tr3) e True Definition	In vitro	Dental wear	Geomagic Qualify 2012 (V.2012_08_08_E, 64-bit, Morrisville, NC, USA)	Intraoral scanning combined with a matching software can accurately quantify clinical wear.
Travassos da Rosa Moreira Bastos et al. (2021) [31]	To investigate the reliability of qualitative tooth wear evaluation through 3D images captured with an IOS and compared to clinical and photographic examinations	Commercial	TRIOS POD	In vivo	Dental wear	N/A	Intraoral scanning seems to be a sound and reliable tool to evaluate tooth wear when compared to traditional methods.
Ayoub et al. (2021) [32]	To assess upper lip scarring and asymmetry in surgically managed unilateral cleft lip and palate (UCLP) cases through 3D images of the nasolabial region	Commercial	TRIOS 3	In vivo	Analysis of the nasolabial region	VRMesh studio VirtualGrid (software version not specified-https://www.vrmesh.com/; accessed on 26 June 2023)	The 3D images are a reliable source for measuring lip asymmetry and the scar surface area.
Michou et al. (2021) [33]	To validate an automated caries scoring system for occlusal caries detection and classification, previously defined for an IOS system featuring fluorescence	Commercial	3Shape TRIOS 4	In vivo	Dental caries	N/A	IOS system proposed exhibits encouraging performance for clinical application on occlusal caries detection and classification.
Ille et al. (2021) [34]	To quantify the feasibility of using an IOS to monitor early erosive tooth wear in patients	Commercial	Planscan Planmeca	In vitro	Dental wear	Geomagic Control X (3Dsystems, Darmstadt, Germany, software version not specified-https://www.3dsystems.com/software; accessed on 26 June 2023)	The intraoral scanner used in this experiment was capable to detect minimal dental tissue loss and could be used to monitor early erosive tooth wear.
Michou et al. (2020) [35]	To develop an automated fluorescence-based caries scoring system for an IOS and to test the performance of the system compared to state-of-the-art methods	Prototype	Prototype (415 nm)	In vitro	Dental caries	N/A	IOS accompanied by an automated caries scoring system may improve objective caries detection and increase the efficiency and effectiveness of oral examinations.
Esquivel-Upshaw et al. (2020) [36]	To test that the dental scanner combined with metrology software will measure clinical wear in vivo in agreement with measurements from X-ray computed microtomography	Commercial	3M True Definition	In vivo	Dental wear	Geomagic Control 2014 (3Dsystems, Darmstadt, Germany-software version not specified-https://www.3dsystems.com/software; accessed on 26 June 2023)	Intraoral scanning combined with a matching software can accurately quantify clinical wear.
Alwadai et al. (2020) [37]	To explore quantitative outcome measures as clinical indicators of simulated occlusal tooth wear progression	Commercial	3M True Definition	In vitro	Dental wear	Rstudio (RStudio, Inc., Boston, MA, USA-software version not specified-https://posit.co/download/rstudio-desktop/; accessed on 26 June 2023) with molaR package	IOS can serve effectively for monitoring overall tooth wear when combined with dental topographic analyses of resultant point clouds.
Michou et al. (2020) [38]	To assess the feasibility of detecting and monitoring early erosive tooth wear using a 3D IOS aided by specific software	Commercial	3Shape TRIOS^®^ 3	In vitro	Dental wear	3Shape TRIOS® Patient Monitoring, version 2.1.1.0	The use of an IOS aided by specific software showed good performance for early detection and monitoring of tooth wear in vitro and has promising potential for in vivo application.
Icen et al. (2020) [39]	To compare the diagnostic accuracy of CBCT units with different voxel sizes with the digital intraoral scanning technique in terms of the detection of periodontal defects.	Commercial	3D Shape TRIOS^®^ Color P13 Shade	In vitro	Periodontal defects	N/A	Smaller voxel sizes and smaller CBCT FOV has the highest sensitivity and diagnostic accuracy.
Kumar et al. (2020) [40]	To investigate the sensitivity of intraoral scanners to quantitatively detect early erosive tooth wear.	Commercial	3M True Definition	In vitro	Dental wear	Geomagic Control (3Dsystems, Darmstadt, Germany-software version not specified-https://www.3dsystems.com/software; accessed on 26 June 2023)	Precision was low, suggesting limitations for minimal changes in tooth wear
Charalambous et al. (2019) [41]	To investigate the threshold and accuracy of intraoral scanning in measuring freeform human enamel surfaces	Commercial	3M True Definition Scanner	In vitro	Dental wear	Geomagic Control (3Dsystems, Darmstadt, Germany-software version not specified-https://www.3dsystems.com/software; accessed on 26 June 2023); Mountains® 8 (Digitalsurf, Besançon, France)	The intraoral scanner had a depth discrimination threshold of 73 µm on unpolished natural enamel and significant differences (*p* < 0.05) were observed compared to NCLP below this level.
Eom et al. (2018) [42]	To evaluate the performance response of a 3D intraoral scan probe based on OCT that enables structural and functional diagnoses of the human teeth	Prototype	Prototipo per OCT	In vitro	Other structural and functional issues	Self-developed multithreaded C++ software, built and compiled in Microsoft Visual Studio 2013.	The feasibility of the intraoral scan probe is demonstrated based on its ability to image and characterize the structure and function of the human teeth.
Deferm et al. (2018) [43]	To assess the feasibility of 3D intraoral scanning for palatal soft tissue analysis.	Commercial	POD TRIOS^®^ 3	In vivo	Soft tissue analysis	Maxilim software (V2.3.0, Medicim NV, Mechelen, Belgium)	IOS can perform a 3D documentation of palatal soft tissue in terms of shape, color, and curvature.

**Table 3:** Summary of the items and categories examined for the excluded papers.

Authors	Aim of the Study	Type of IOS	Product Name	Type of Study	Application	Main Results
Ferraro et al. (2022) [44]	To compare the accuracy of 3D printed models fabricated from CBCT scans of human mandibular dry skulls with models derived from IOSs.	Commercial	TRIOS	In vitro	Comparison of 3D model technical parameters in reconstruction	Differences not clinically detectable for orthodontic applications
Akyalcin et al. (2018) [45]	To evaluate the accuracy of 3D digital models acquired from a chairside IOS compared with both manual and CBCT measurements of the same dental anatomy.	Commercial	Cadent iTero	In vitro	Comparison of 3D model technical parameters in reconstruction	Differences not clinically detectable for orthodontic applications
Fraile et al. (2022) [46]	To compare the interocclusal contact records obtained by three different digital methods with the conventional method (articulating paper).	Commercial	TRIOS Color POD	In vivo	Comparison of 3D model technical parameters in reconstruction	Reliable performance of the scanners compared to the gold standard
Kirschneck et al. (2018) [47]	To assess the reliability, validity, and conformity of an intraoral scanning procedure and two extraoral digitization workflows via alginate impression and plaster model scanning.	Commercial	Lythos	In vivo	Comparison of 3D model technical parameters in reconstruction	Better reliability of the extraoral 3D models.
Davidovich et al. (2020) [48]	To present an innovative treatment approach for endodontically treated teeth in children using a digital workflow with IOS and CAD/CAM fabrication of the restoration	Commercial	Primescan connect	In vivo	Endodontically treatment approach	The high accuracy of the scanner enables definitive restoration in young patients
Davidovich et al. (2020) [49]	To present an innovative treatment approach for children with MIH using a digital workflow with IOS and CAD-CAM fabrication of the restoration	Commercial	Primescan connect	In vivo	Treatment for molar incisor hypomineralization (MIH)	The digital workflow provides definitive restorations in young patients due to the high accuracy of the scanning.
Revilla-Leon et al. (2019) [50]	To evaluate the influence of the interocclusal space on the accuracy of the maxillomandibular relationship captured with an IOS.	Commercial	TRIOS 4	In vitro	Fabrication of dental prostheses	The interocclusal space available when acquiring virtual bilateral occlusal records using the tested IOS impacted the accuracy of the maxillomandibular relationship.
Revilla-Leon et al. (2020) [51]	To describe a digital workflow for planning an esthetic treatment by using a facial and intraoral scanner, the dental and open-source software design of a facially generated diagnostic waxing, and additive manufactured (AM) clear silicone indices.	Commercial	iTero Element	In vivo	Esthetic rehabilitation	Three-piece AM clear indexes provided advantages compared with conventional procedures
Park et al. (2020) [52]	To describe a digital workflow protocol for treatment planning of an esthetic rehabilitation using direct composite restorations	Commercial	TRIOS 3	In vivo	Esthetic rehabilitation	Provides several advantages compared with conventional procedures.
DuVall (2021) [53]	To describe a procedure that uses an intraoral scanner and milling unit to fabricate a chairside computer-aided design and computer-aided manufacturing radiographic and surgical guide for use with a Cone Beam Computed Tomography system	Commercial	CEREC Omnicam AC	In vivo	Dental implants	The procedure described is efficient compared with the traditional one.
Ahmed et al. (2020) [54]	To describe an approach to manage excessive gingival display by lengthening of the clinical crowns using a digital workflow.	Commercial	TRIOS 3	In vivo	Surgical digital procedure	The procedure described is efficient compared with the traditional one.

**Table 4:** Main features and technical characteristics of commercial IOSs.

Scanner Model	Year	Price	Technology of Acquisition	Illumination	Wand Dimensions [mm]	Wand Weight [g]
TRIOS 3 (3Shape)	2015	$18,950.00	Structured light	LED	N/A	340
TRIOS 4 (3Shape)	2019	$30,000.00	Structured light + AI scan	LED	274 × 42 × 12	345
TRIOS 5 (3Shape)	2022	$29,500.00	Structured light + AI	N/A	266 × 38 × 11	N/A
True Definition (3M Espe)	2012	$12,000.00	Structured Light	Blue	254 × 16 × 14	233
iTero Element (Align Technology)	N/A	N/A	N/A	Red laser light (680 nm) and white LED	38.5 × 53.5 × 69.8	470
iTero (Align Technology)	2007	N/A	parallel confocal	white LED light	N/A	680.39
iTero 5D Element (Align Technology)	2021	N/A	Structured Light + NIRI	N/A	N/A	~500
CS3800 (Carestream)	2021	$38,000.00	N/A	Red, Green, Blue LEDs	226 × 38 × 60	240
CS3700 (CareStream)	N/A	$16,000.00	N/A	N/A	N/A	N/A
CS3600 (CareStream)	2016	$37,000.00	Structured Led Light	Amber, Green, Blue LEDs	220 × 38 × 58	325
CS3500 (CareStream)	2017	N/A	Parallel confocal	N/A	N/A	N/A
Virtuo Vivo (Dental wings)	2019	$19,000.00	Multiscan Imaging Technology	Blue laser	200 × 30	120
DWIO (Dental Wings)	2017	N/A	Multiscan Imaging	Blue LED	N/A	105
IOS FastScan (Glidewell Laboratories)	N/A	$19,899.00	Active triangulation/Scheimpflug principle	N/A	N/A	N/A
i700 (Medit)	2021	$20,000.00	3D-in-motion video technology	LED	248 × 44 × 47.4	245
i500 (Medit)	2020	$20,000.00	3D-in-motion video technology	Blue, White	266 × 18 × 15.2	276
3D Progress (MHT S.p.A.)	N/A	N/A	Confocal microscope + Moireé effect detection	N/A	N/A	N/A
Lythos (Ormco)	2013	N/A	Accordion fringe interferometry	N/A	N/A	317.51
Planscan (Planmeca)	N/A	N/A	Triangulation	N/A	N/A	N/A
Emerald (Planmeca)	N/A	N/A	N/A	Laser	41 × 45 × 249	183
Aoralscan 3 (Shining 3D)	2021	$15,200.00	Structured light	N/A	281 × 33 × 46	240
Cerec Omnicam (Dentsply Sirona)	2012	$35,000.00	Optical triangulation and confocal microscopy	White LED	228 × 16 × 16	N/A
Cerec AC BlueCam (Dentsply Sirona)	2009	N/A	confocal microscopy/active triangulation	Blue LED	N/A	N/A
Cerec PrimeScan (Dentsply Sirona)	2019	$26,000.00	High-frequency optical contrast analysis	N/A	N/A	457
3Di IOS (MyRay)	N/A	$29,500.00	Active Stereo Imaging	N/A	256 × 45 × 45	150
3DISC (Heron IOS)	N/A	N/A	N/A	N/A	N/A	145

N/A: Not Available.

**Table 5:** Main IOSs used for diagnostic applications.

Diagnostic Application	Intraoral Scanners
Periodontal defects	3D Shape TRIOS^®^
Soft tissue analysis	3Shape TRIOS^®^ 3
Analysis of the Nasolabial Region	3Shape TRIOS^®^ 3
Orthodontic diagnosis	iTero
CBCT improvement	Carestream CS3600/Medit i700
Other structural and functional issues	prototype for OCT

## Data Availability

Data can be made available upon reasonable request to the corresponding author.
